# Antibiotic Resistance Pattern and Surgical Outcome in Complicated Intra-Abdominal Infections Due to Colorectal Perforation

**DOI:** 10.3390/antibiotics15020147

**Published:** 2026-02-02

**Authors:** Jacopo Giuliani, Camilla Cremonini, Serena Musetti, Giuseppe Zocco, Ismail Cengeli, Dario Tartaglia, Massimo Chiarugi, Alice Salamone, Ettore Melai, Francesco Forfori, Benedetta Tuvo, Iacopo Franconi, Antonella Lupetti, Lorenzo Ghiadoni, Federico Coccolini

**Affiliations:** 1 General, Emergency and Trauma Surgery Department, Pisa University Hospital, 56124 Pisa, Italy; 2 ICU Department, Pisa University Hospital, 56124 Pisa, Italy; 3 Microbiology Department, Pisa University Hospital, 56124 Pisa, Italy; 4 Emergency Medicine Department, Pisa University Hospital, 56124 Pisa, Italy

**Keywords:** antibiotic resistance, surgical outcomes, intra-abdominal infections, bacterial epidemiology, microbiological sample, microbiological culture, polymicrobial infections

## Abstract

**Background:** Intra-abdominal infections (IAIs) are one of the leading causes of non-traumatic death in emergency surgery units. The appropriateness of empirical antibiotic therapy is fundamental for outcomes and for limiting the spread of resistance. This study aimed to assess the epidemiology and antibiotic resistance patterns of microorganisms recovered from complicated intra-abdominal infections due to colorectal perforation at an Italian University Hospital during a nine-year period. **Methods**: This study evaluated a cohort of patients subjected to emergency surgery for colonic perforation with collected intrabdominal fluid samples from 2015 to 2024. Patterns of isolated bacteria and antibiotic resistance status were collected and correlated to patient outcomes. **Results**: 321 patients were enrolled; the average age was 70.2 years. The main diagnoses were complicated diverticulitis (58%), colorectal carcinoma perforation (18%), and acute intestinal ischemia (24%). 80.4% were immunocompromised; average hospital stay (HLOS) was 15.6 days; 60.1% developed postoperative complications. Microbiological cultures were available for 111 patients: 56.7% had mono-microbial infections and 43.3% multi-microbial infections. 53 antibiotics and 9 antifungals were tested, with resistance rates exceeding 20% for many pathogens. Multivariate analyses showed that documented IAIs are associated with longer postoperative hospital stays (p 0.003 CR 8.075) but not with patient mortality (p 0.031). Prolonged HLOS was more commonly observed in patients with polymicrobial infections or infections caused by multi-drug-resistant organisms (p 0.03; p 0.003). **Conclusions**: Microbiological characteristics of isolated bacteria do not directly influence mortality; however, the presence of polymicrobial infections and resistant pathogens directly affects the duration of hospitalization and often leads to the development of chronic disease conditions.

## 1. Introduction

Intra-abdominal infections (IAIs) are common surgical emergencies and represent one of the leading causes of non-traumatic death in emergency surgery units worldwide. These infections comprise a broad and heterogeneous group of conditions characterized by an infectious process involving an abdominal organ or tissue [1]. The infection may be confined to the abdominal organ of origin (uncomplicated intra-abdominal infection) or extend to adjacent peritoneal or retroperitoneal structures or the peritoneal cavity itself (complicated intra-abdominal infections).

In uncomplicated forms, the infection remains confined within an organ, with no anatomical alterations of the gastrointestinal tract and without breaching the organ barrier. In complicated forms, the disease extends beyond the visceral peritoneum into the peritoneal space, resulting in abscess formation and/or peritonitis, which may be localized or diffuse [2]. These are severe clinical conditions that can involve diffuse peritoneal inflammation and carry the risk of progression to sepsis, septic shock, multi-organ failure, and death, with a reported global mortality rate of 9.2%. Despite a reduction in mortality over the past 15–20 years due to the implementation of early diagnosis and intensive care support, many patients today develop chronic critical illness, resulting in prolonged hospital stays, high resource utilization, and frequent discharge to post-acute care facilities.

Treatment for these conditions is based on three fundamental pillars: early initiation of antibiotic therapy, effective anatomical and physiological source control, and adequate fluid resuscitation and supportive care, especially in patients presenting with sepsis or septic shock [1]. Without an aggressive approach, there is a risk of progression to sepsis, septic shock, multi-organ dysfunction, and death [3,4,5,6].

“Source control” is the core of treatment: without it, the effectiveness of antibiotic therapy is limited, mortality rates can reach 100% and stabilizing the patient becomes challenging. During source control, the collection of samples from peritoneal fluid or abscesses for microbiological analysis (culture and antibiotic susceptibility testing) is essential to optimize antibiotic therapy [3,7,8].

The appropriateness of antibiotic therapy is crucial in determining clinical outcomes in these patients. Inappropriate antibiotic therapy is associated with prolonged hospital stays and increased mortality [1,3]. Antibiotic therapy must be initiated early and is therefore empirical in its initial phase [9]. Drug selection at this stage is guided by microbiological epidemiology, including the expected pathogens and patterns of antibiotic resistance [10,11,12,13]. Consequently, monitoring antibiotic resistance patterns is essential for appropriate therapeutic decisions [3].

The prevalence of antibiotic-resistant bacterial infections is steadily increasing, presenting a major challenge in antimicrobial therapy and a significant global health issue [1].

The use of adequate antibiotics is critical not only for individual patient outcomes, where it determines treatment efficacy, but also at the population level, as it minimizes the spread of antibiotic-resistant microorganisms. Optimizing empirical probabilistic antibiotic therapy and adjusting it based on culture results aligns with the principles of antibiotic stewardship. Once culture results are available (from intra-abdominal or blood samples), the antibiotic regimen can be adjusted, either by broadening or narrowing the spectrum of activity [1,14,15,16].

Antibiotic stewardship programs aim to develop guidelines for antibiotic use that address the growing challenge of antibiotic resistance [17,18,19].

The optimization of antibiotic use does not end with the selection of the most appropriate drug but also encompasses numerous other aspects: in 2023, the Global Alliance for Infections in Surgery (GAIS) [17] developed a ten-rule guideline for appropriate antimicrobial use in hospitals.

This study is therefore designed with the intention of implementing the knowledge of local bacteria epidemiology that may potentially improve the surveillance and monitoring of antibiotic resistance and the selection of the correct antibiotic.

## 2. Results

### 2.1. Patients’ Characteristics

**Figure 1:**
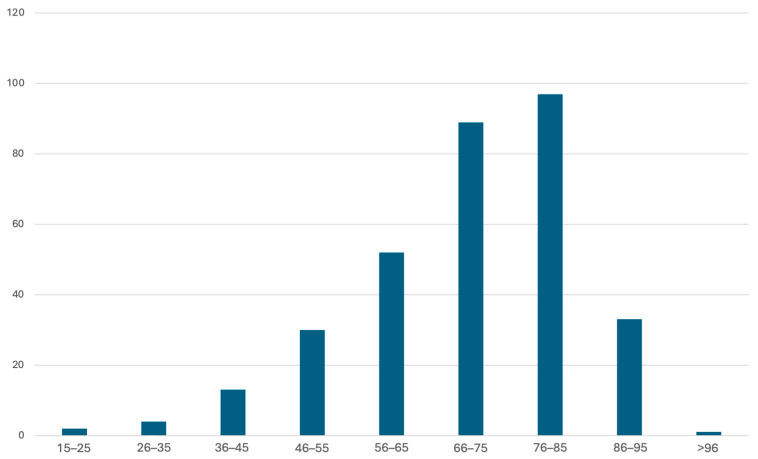
Sample age distribution.

A total of 321 patients were enrolled in the analysis. 136 (42.4%) were males and 185 (57.6%) females. The mean age was 70.2 years, with a median age of 73 years (SD ± 14.1) (Figure 1).

Out of the 321 analysed patients, 118 (58%) had a diagnosis of complicated acute diverticulitis, 57 (18%) had colorectal carcinoma perforation, and 76 (24%) presented with intestinal perforation due to acute bowel ischemia.

**Figure 2:**
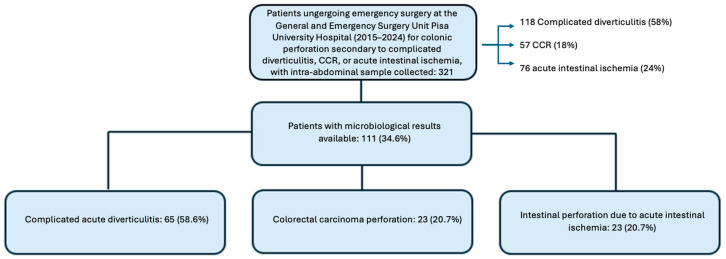
Sample distribution.

Microbiological analyses were available for a total of 111 patients, including 65 (58.6%) with complicated acute diverticulitis, 23 (20.7%) with colorectal carcinoma perforation, and 23 (20.7%) with intestinal perforation due to acute intestinal ischemia (Figure 2).

Among the sample, 63 patients (19.6%) were immunocompetent, while 258 patients (80.4%) had some degree of immunosuppression. Within the immunosuppressed group, 254 (98%) met the criteria for moderate immunosuppression, and 53 (20.5%) fulfilled the criteria for severe immunosuppression.

The average LOS was 15.6 days (median 12 days, SD ± 13.3), with a mean postoperative course duration of 13.8 days (median 10 days, SD ± 12.7 days).

During the postoperative period, 196 patients (60.1%) experienced various postoperative surgical complications with varying severity (Calvien–Dindo scores ≥ 2), which were distributed as follows: 91 (46.4%) with score 2, 26 (13.2%) with score 3, 11 (5.6%) with score 4, 67 (34.2%) with score 5 (death).

Out of the total cohort, 244 (70.0%) patients were discharged, while 67 (20.9%) patients died during hospitalization.

In-hospital mortality varied significantly across the three disease groups: Complicated diverticulitis: 21 deaths among 118 patients (17.7%); colorectal carcinoma perforation: 9 deaths among 57 patients (15.7%); intestinal perforation due to acute intestinal ischemia: 37 deaths among 76 patients (48.7%).

Among the 67 patients who died postoperatively, 46 (68.7%) died during their stay in the intensive care unit (ICU), while the remaining 13 (19.4%) died in the general ward.

Of the 244 discharged patients 196 (80.3%) were discharge at home; 19 (7.8%) were transferred to post-acute care or rehabilitation facilities and 24 (9.8%) were transferred to other departments for further treatments.

### 2.2. Microbiological Analysis and Antimicrobial Susceptibility

Microbiological positivity was observed in 111 (34.6%) out of 321 patients. In this subgroup, 63 patients (56.7%) exhibited mono-microbial cultures, while 48 patients (43.3%) had multi-microbial cultures. The culture examinations performed on the collected samples identified 29 distinct bacterial/fungal species. A detailed distribution of the various bacterial and fungal species is presented in Table 1.

The microbiological samples were tested for antimicrobial susceptibility. A total of 53 antibiotics and 9 antifungal agents were evaluated. The percentages of antibiotic resistance for the various isolated genera are presented in, highlighting antibiotic resistance rates exceeding 20%.

**Figure 3:**
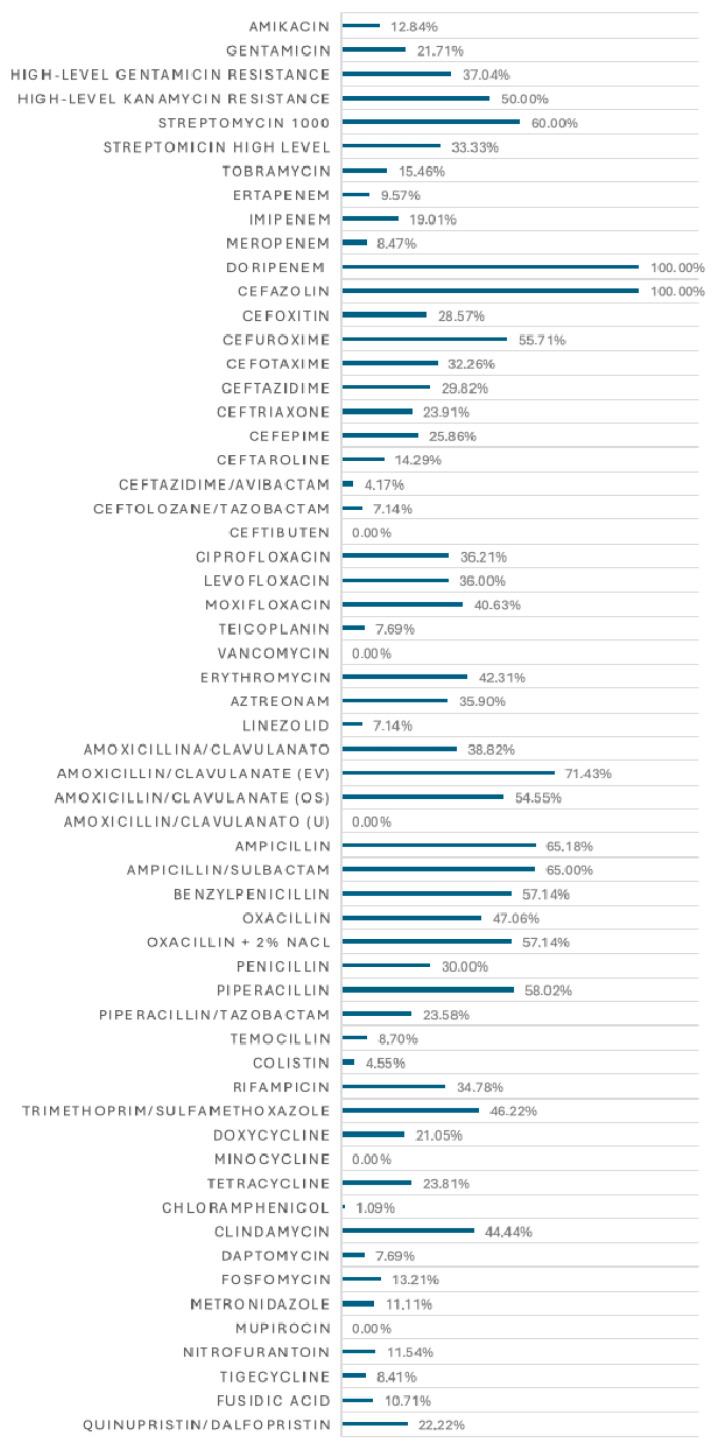
Cumulative antibiotic resistance rates on the total number of isolates tested for each antibiotic.

Cumulative antibiotic resistance rates were calculated based on the total number of isolates as well as on the total number of isolates tested for each antibiotic, as shown in Figure 3.

Antifungal resistance rates are reported separately for *C. albicans* and other *Candida* species. This distinction was made due to the significantly higher prevalence of *C. albicans.*

### 2.3. Clinical Outcome Analysis

The results of the multivariate analysis concerning the LOS (Table 2) show a statistically significant correlation between the type of diagnosis and the duration of hospitalization (*p* < 0.001). Moreover, a statistically significant correlation was observed between prolonged hospitalization and the presence of resistant and multidrug-resistant (MDR) organisms (*p* = 0.011; CR = 6.780).

The results of the multivariate analysis regarding the duration of the postoperative course (Table 3) confirm a positive correlation between a longer postoperative course and the presence of MDR organisms in the microbiological isolates (*p* = 0.003; CR = 8.075). In addition, a statistically significant association was found between prolonged postoperative recovery and the presence of multiple pathogens isolated from the same patient’s microbiological sample (*p* = 0.030; CR = 3.278).

The multivariate analysis regarding in-hospital mortality (Table 4) demonstrates a significant statistical correlation between mortality and patient age (*p* < 0.031), with older patients exhibiting a worse prognosis. In-hospital mortality was also significantly correlated with a diagnosis of intestinal ischemia (*p* < 0.001), indicating that the type of intra-abdominal infection influences patient prognosis due to the distinct pathophysiological alterations associated with sepsis.

An unexpected finding in this study was that mortality did not correlate with the characteristics of the analysed microbiological samples, including the number of isolates, the presence of resistant organisms, or the presence of MDR organisms.

The multivariate analysis concerning the occurrence of postoperative complications (Table 5) did not reveal any statistically significant correlation with the patient’s characteristics or the microbiological culture findings under investigation. No single factor appeared to have a predominant influence on the development of a complicated postoperative course.

## 3. Discussion

The sample distribution by sex and age group reflects the epidemiology of complicated IAI, with a peak prevalence between 75–85 years and a higher incidence in females [20,21,22]. Complicated acute diverticulitis accounts for more than half of the cases, while colorectal carcinoma perforation and intestinal ischemia represent 1/5 and 1/4 of the sample, respectively. This distribution is attributable to the higher prevalence of complicated acute diverticulitis in the general population (approximately 50% of colorectal perforation cases are diverticular) compared to colorectal carcinoma perforation and intestinal ischemia (particularly colonic ischemia), which are inherently less frequent events [23].

In the analysed sample, the percentage of patients with an immunocompromised state exceeds that of the general population [24]. This observation is influenced by the sample’s characteristics: the age of patients, the presence of active neoplastic disease, associated therapies and comorbidities [25]. The conditions under study, in fact, affect a population that is generally in the later decades of life and thus reflects the impact of associated comorbidities.

The most frequently isolated bacteria/yeast belonged to the normal intestinal flora, including *Escherichia coli*, *Enterococci*, *Candida albicans*, *Enterobacteriaceae*, and *Citrobacter freundii* [26]. *Staphylococcus* and *Streptococcus* species were also isolated, as well as *Serratia marcescens*, though less commonly. *Pseudomonas aeruginosa* and *Klebsiella pneumoniae* emerged as important pathogens, particularly in immunocompromised patients or those with prior antibiotic therapy [26]. While the association between infections by *K. pneumoniae* or *P. aeruginosa* and previous antibiotic administration was not specifically evaluated, colonization by these pathogens in hospitalized patients [26,27] may influence the selection of empirical antibiotic therapy.

The description of antibiotic resistance rates for the isolated pathogens provides crucial insights into the local and regional microbiological epidemiology. This information must be considered when establishing empirical antibiotic therapy for patients with complicated intra-abdominal infections.

*Enterobacteriaceae*, including *E. coli*, *Klebsiella* spp., *Enterobacter* spp., and *C. freundii*, exhibited resistance rates to penicillins exceeding 20%, except for piperacillin–tazobactam (40% resistance in *Klebsiella* spp. and *Citrobacter* spp.). For second- and third-generation cephalosporins, resistance rates generally exceeded 20%, limiting the use of these drugs in empirical therapy. However, newer cephalosporins, such as ceftazidime–avibactam and ceftolozane–tazobactam, displayed lower resistance rates, making them viable therapeutic options. *Klebsiella* spp. also showed resistance to carbapenems, aminoglycosides, and fluoroquinolones.

Significant resistance was observed among Gram-positive cocci, including Enterococci, Staphylococci, and Streptococci, with resistance rates exceeding 20% for beta-lactams and fluoroquinolones. Nevertheless, no isolates exhibited resistance to tigecycline.

*Pseudomonas aeruginosa* showed notable resistance to imipenem, ceftazidime, cefepime, and piperacillin–tazobactam (29% resistance), as well as to fluoroquinolones such as ciprofloxacin and levofloxacin (24% and 41%, respectively). These findings are critical given the increasing prevalence of resistance among nosocomial bacteria [28].

Drugs like amoxicillin/clavulanate and amoxicillin displayed resistance rates exceeding 20%, limiting their use. However, the piperacillin–tazobactam combination retained good efficacy, though it is classified under the “Watch” category in the AWaRe classification [29]. Cephalosporins such as ceftazidime, cefazolin, and cefoxitin had resistance rates below 20%, while other third-generation cephalosporins exhibited resistance rates above 20%. The use of ceftazidime–avibactam and ceftolozane–tazobactam should be avoided in empirical therapy as they are classified as “Reserve” antibiotics in the AWaRe classification [29,30]. Aminoglycosides, such as amikacin and gentamicin, remain important therapeutic options but must be used cautiously due to their side effects.

Doxycycline and nitrofurantoin, with resistance rates below 20%, may represent interesting therapeutic alternatives. Doxycycline offers a broad spectrum of activity, and nitrofurantoin has historically been used for urinary tract infections [31,32]. These drugs could potentially be useful in complicated intra-abdominal infections, although further studies are necessary.

The prevalence of *C. albicans* (13.04%) aligns with the (%) observed in previous studies [33], whereas all *C. albicans* isolates were susceptible to fluconazole in this study. Azole resistance was detected particularly in *C. glabrata* and *C. parapsilosis,* with the latter showing rates of fluconazole resistance above 50% of isolates. Nevertheless, echinocandins remain the appropriate therapeutic choice for intra-abdominal fungal infections [33,34].

The analysis of clinical outcomes in patients undergoing surgery for complicated intra-abdominal infections highlights the influence of the underlying diagnosis on hospital stay duration and mortality. Patients with complicated acute diverticulitis and colorectal perforation exhibited longer hospital stays compared to those with intestinal necrosis, primarily due to the higher perioperative mortality rate in the latter group. This reflects the combined impact of ischemic and septic conditions, often resulting in shorter yet unfavorable hospital courses.

Moreover, the isolation of antibiotic-resistant or multi-drug-resistant (MDR) bacteria from microbiological samples correlates with longer hospital stays and extended postoperative courses. Multivariate analysis confirms that the presence of MDR bacteria is a determinant of prolonged hospitalization. Immunosuppressed patients also tend to have extended postoperative recoveries. These findings suggest that the increasing prevalence of antibiotic resistance complicates the management of intra-abdominal infections, with significant implications for healthcare costs.

Mortality within the examined sample was higher than reported in the literature, likely due to the inclusion of only patients with complicated infections [1]. Advanced age was associated with increased in-hospital mortality, with a higher mean age of 75 years observed in deceased patients compared to 68 years in those discharged. Multivariate analysis confirms this correlation. Although immunosuppression appeared to influence postoperative mortality, this relationship was not significant in the multivariate analysis. Mortality due to intestinal necrosis from acute ischemia (48%) aligns with the literature (50%) [35], suggesting that the underlying pathology and its associated factors significantly impact patient survival.

Unlike other variables, in-hospital mortality did not appear to be influenced by microbiological findings, such as antibiotic resistance or polymicrobial infections, indicating that current treatment protocols remain effective. The lack of association between microbiological resistance patterns and mortality rates can partly be attributed to the limitations of our current diagnostic techniques. Standard lab methods, like EUCAST or CLSI, usually only examine a tiny fraction of the total bacterial population at the infection site. As pointed out by Andersson et al. (2019) [36], these techniques might miss important issues like heteroresistance or the existence of small multi-drug-resistant (MDR) subpopulations. These undetected resistant strains could have a major impact on clinical outcomes and the effectiveness of empirical therapy, even if the overall isolate seems susceptible in vitro.

Despite the complexity of intra-abdominal infections, advancements in surgical techniques and antibiotic management have contributed to reducing mortality over time [1].

Postoperative complications were positively correlated with immunosuppression, advanced age, and the presence of resistant bacteria. However, multivariate analysis did not identify a significant relationship between these factors and postoperative morbidity. The lack of specific data on complications limits the interpretation of these findings.

Most of the patients (196–80.3%) were discharged at home, while some were transferred to rehabilitation facilities or other departments (43–17.6%).

The study has several limitations, including patient selection and its retrospective design, which hindered the collection of critical data such as intensive care unit stay duration and details of antibiotic treatments. Additionally, the heterogeneity of the sample across different diseases (acute diverticulitis, carcinoma perforations, and intestinal ischemia) represents another potential limitation. Lastly, it should be noted that the sample does not represent all treated patients but only a subset due to the inclusion only of those who had complete microbiological analysis. A study limitation that must be disclosed lies within the current laboratory routine workflow and the retrospective nature of the study. Authors acknowledge the important limitations of antimicrobial susceptibility testing via broth microdilution test which to date is still the gold standard in detecting resistant phenotypes, however in a broader and deeper view in terms of antimicrobial resistance, molecular methods along with whole genome sequencing show the highest sensitivity in depicting and deciphering underlying antimicrobial resistant patterns within the study population, as they can evaluate heteroresistant and tolerance genomic traits within the biological diversity present in the patient.

## 4. Methods

This study retrospectively analyzed data of patients who underwent emergency surgery at the General and Emergency Surgery Unit at Pisa University Hospital, Italy, between January 2015 and August 2024, with diagnoses of colonic perforation due to complicated acute diverticulitis, colorectal carcinoma, or acute intestinal ischemia, for whom intraoperative intra-abdominal cultures were available. Clinical data were extracted from the hospital electronic medical records. Microbiological results from these samples, including peritoneal fluid, blood, and other specimens (urine, broncho-aspirate, surgical wound swabs), were obtained from the microbiology department.

The analyses included culture tests, microbial species identification, and susceptibility testing via microbroth dilution, assessed based on minimum inhibitory concentration (MIC) values according to EUCAST guidelines (SIR system: sensitive, susceptible, increased exposure—previously defined as “intermediate susceptibility”—and resistant) [37]. Isolates of *Candida* spp. were interpreted following CLSI standards [38]. Sensititre YeastOne Y010© (Thermo Fisher Diagnostics B.V., Lansmeer, The Netherlands) was used for antifungal susceptibility testing. Yeast isolates were incubated at 37 °C for 24–48 h under aerobic conditions, then 3 to 5 separeted yeast colonies were suspended in sterile distilled water. The solution was vortexed and homogenized to reach a final concentration of 0.5 McFarland analyzed with a spectrophotometer. The final inoculum concentration was 0.5 × 10^3^–2.5 × 10^3^ cells/mL according to the CLSI guidance document. Colorimetric broth microtitration plate Sensititre YeastOne Y010© was incubated at 35–37 °C and checked for growth of the fungal microorganism at 24 h and 48 h according to different species.

Antibiotics with intermediate sensitivity were classified as “non-resistant” for calculating resistance percentages, which were based on the total number of microorganisms tested.

Immunocompromised status was defined according to the parameters established by the World Society of Emergency Surgery (WSES) [39].

Clinical outcomes analysed included in-hospital mortality, length of stay (LOS), postoperative course, and postoperative morbidity, defined as a complicated postoperative course (Clavien–Dindo score ≥ 2) [40]. Length-of-stay data were retrieved from clinical records in the electronic database. Missing data were considered as absent (missing) for analysis. The study protocol (n. 17575) was approved by the Regional Ethical Committee. The statistical analysis included the description of sample characteristics, microbiological epidemiology, and the association between clinical outcomes, immunosuppression, and microbiological patterns of isolates. Categorical data were described using absolute and relative frequencies (%), while continuous variables were expressed as mean and standard deviation (SD). Associations between qualitative variables were analysed using the chi-square test; for quantitative variables, *t*-tests or ANOVA were employed. Pearson’s correlation analysis was used for quantitative outcomes. Multivariate analysis was conducted using logistic regression models for categorical outcomes and linear regression models for quantitative outcomes, with statistical significance set at 0.05. Analyses were performed using SPSS version 29.

## 5. Conclusions

Microbiological characteristics of isolated bacteria do not directly influence mortality; however, the presence of polymicrobial infections and resistant pathogens directly affects the duration of hospitalization and often leads to the development of chronic disease conditions.

Despite its limitations, the study gives strong support to the necessity of continuously monitoring antibiotic resistance patterns to optimize empirical therapy selection for complicated intra-abdominal infections. Antibiotic resistance constrains the efficacy of many commonly used antibiotics, while identifying drugs with low resistance rates could guide future research in empirical therapy.

## Figures and Tables

**Table 1:** Distribution of the various bacterial/yeast species.

**Escherichia**		66	35.87%
	*Escherichia coli*	66	35.87%
**Candida**		29	15.76%
	*Candida albicans*	24	13.04%
	*Candida glabrata*	2	1.09%
	*Candida norvegensis*	1	0.54%
	*Candida parapsilosis*	2	1.09%
**Enterococcus**		22	11.96%
	*Enterococcus avium*	2	1.09%
	*Enterococcus faecalis*	7	3.80%
	*Enterococcus faecium*	13	7.07%
**Klebsiella**		18	9.78%
	*Klebsiella oxytoca*	2	1.09%
	*Klebsiella pneumoniae*	4	2.17%
	*Klebsiella pneumoniae* spp. *pneumoniae*	12	6.52%
**Pseudomonas**		17	9.24%
	*Pseudomonas aeruginosa*	17	9.24%
**Staphylococcus**		13	7.07%
	*Staphylococcus aureus*	2	1.09%
	*Staphylococcus epidermidis*	7	3.80%
	*Staphylococcus haemolyticus*	4	2.17%
	*Saphylococcus lugdunensis*	1	0.54%
**Streptococcus**		6	3.26%
	*Streptococcus anginosus*	2	1.09%
	*Streptococcus constellatus*	1	0.54%
	*Streptococcus gallolyticus*	1	0.54%
	*Streptococcus parasanguinis*	1	0.54%
	*Streptococcus salivarius*	1	0.54%
**Enterobacter**		3	1.63%
	*Enterobacter aerogenes*	1	0.54%
	*Enterobacter cloacae*	1	0.54%
	*Enterobacter cloacae complex*	1	0.54%
**Serratia**		3	1.63%
	*Serratia marcescens*	3	1.63%
**Acinetobacter**		2	1.09%
	*Acinetobacter baumannii*	2	1.09%
**Citrobacter**		2	1.09%
	*Citrobacter freundii*	2	1.09%
	*Citrobacter koseri*	1	0.54%
**Stenotrophomonas**		1	0.54%
	*Stenotrophomonas maltophilia*	1	0.54%

**Table 2:** Results of the multivariate analysis concerning the LOS.

	CR	IC 95%-Inf	IC 95%-Sup	r Parziale	*p*-Value
**Infection category: (1) Diverticulitis, (2) UCR, (3) Intestinal ischemia**	−4.566	−6.198	−2.935	−0.285	<0.001
**Positive cultures: (0) no, (1) yes**	−2.938	−10.791	4.914	−0.104	0.462
**At least 1 resistant: (0) no, (1) yes**	−3.284	−9.076	2.507	−0.107	0.265
**At least 1 MDR: (0) no isolated, (1) no MDR, (2) MDR**	6.780	1.584	11.976	0.372	0.011
**Number of bacterial species in microbiological sample**	1.641	−1.091	4.372	0.116	0.238
*Intercept of the linear model*	19.631	16.438	22.823		<0.001

**Table 3:** Results of the multivariate analysis regarding the duration of the postoperative course.

	CR	IC 95%-Inf	IC 95%-Sup	r Parziale	*p*-Value
**Immunosuppression: (0) no, (1) yes**	2.642	−0.808	6.091	0.081	0.133
**Positive cultures: (0) no, (1) yes**	−3.749	−11.631	4.133	−0.140	0.350
**At least 1 resistant: (0) no, (1) yes**	−5.199	−10.870	0.472	−0.176	0.072
**At least 1 MDR: (0) no, (1) yes**	8.075	2.832	13.318	0.460	0.003
**Number of bacterial species in microbiological sample**	3.278	0.312	6.245	0.235	0.030
*Intercept of the linear model*	8.529	5.337	11.722		<0.001

**Table 4:** Results of the multivariate analysis regarding in-hospital mortality.

	CR	OR	IC 95%-Inf	IC 95%-Sup	*p*-Value
**Complicated diverticulitis: (0) no, (1) yes**	−0.087	0.917	0.369	2.281	0.852
**Intestinal ischemia: (0) no, (1) yes**	1.977	7.224	2.961	17.628	<0.001
**Immunocompromisation mild and moderate: (0) no, (1) yes**	−0.060	0.942	0.211	4.197	0.937
**Age**	0.032	1.032	1.003	1.062	0.031
**Immunocompromisation mild and moderate TOTAL**	0.225	1.252	0.759	2.066	0.379
*Intercept of the linear model*	−4.409	0.012			<0.001

**Table 5:** Results of the multivariate analysis concerning the occurrence of postoperative complications.

	CR	OR	IC 95%-Inf	IC 95%-Sup	*p*-Value
**Gender: (0) M, (1) F**	0.400	1.492	0.889	2.505	0.130
**Complicated diverticulitis: (0) no, (1) yes**	−0.342	0.710	0.403	1.251	0.236
**Immunocompromisation mild and moderate: (0) no, (1) yes**	0.687	1.988	0.729	5.421	0.180
**Immunocompromisation severe: (0) no, (1) yes**	−0.806	0.447	0.040	4.941	0.511
**Positive cultures: (0) no, (1) yes**	0.537	1.712	0.334	8.759	0.519
**At least 1 resistant: (0) no, (1) yes**	−0.784	0.456	0.138	1.509	0.199
**At least 1 MDR: (0) no, (1) yes**	0.537	1.712	0.591	4.958	0.322
**Age**	0.022	1.022	0.998	1.046	0.070
**Immunocompromisation mild and moderate TOTAL**	0.216	1.242	0.772	1.998	0.373
**Immunocompromisation severe TOTAL**	1.164	3.201	0.406	25.221	0.269
**Number of bacterial species in microbiological sample**	0.241	1.273	0.722	2.245	0.405
*Intercept of the linear model*	−2.313	0.099			0.002

## Data Availability

The datasets generated during and/or analysed during the current study are available from the corresponding author on reasonable request.
